# Dynamic Partnership between TFIIH, PGC-1α and SIRT1 Is Impaired in Trichothiodystrophy

**DOI:** 10.1371/journal.pgen.1004732

**Published:** 2014-10-23

**Authors:** Hussein Traboulsi, Serena Davoli, Philippe Catez, Jean-Marc Egly, Emmanuel Compe

**Affiliations:** Institut de Génétique et de Biologie Moléculaire et Cellulaire, Illkirch, Strasbourg, France; École Polytechnique Fédérale de Lausanne (EPFL), Switzerland

## Abstract

The expression of protein-coding genes requires the selective role of many transcription factors, whose coordinated actions remain poorly understood. To further grasp the molecular mechanisms that govern transcription, we focused our attention on the general transcription factor TFIIH, which gives rise, once mutated, to Trichothiodystrophy (TTD), a rare autosomal premature-ageing disease causing inter alia, metabolic dysfunctions. Since this syndrome could be connected to transcriptional defects, we investigated the ability of a TTD mouse model to cope with food deprivation, knowing that energy homeostasis during fasting involves an accurate regulation of the gluconeogenic genes in the liver. Abnormal amounts of gluconeogenic enzymes were thus observed in TTD hepatic parenchyma, which was related to the dysregulation of the corresponding genes. Strikingly, such gene expression defects resulted from the inability of PGC1-α to fulfill its role of coactivator. Indeed, extensive molecular analyses unveiled that wild-type TFIIH cooperated in an ATP-dependent manner with PGC1-α as well as with the deacetylase SIRT1, thereby contributing to the PGC1-α deacetylation by SIRT1. Such dynamic partnership was, however, impaired when TFIIH was mutated, having as a consequence the disruption of PGC1-α recruitment to the promoter of target genes. Therefore, besides a better understanding of the etiology of TFIIH-related disease, our results shed light on the synergistic relationship that exist between different types of transcription factors, which is necessary to properly regulate the expression of protein coding genes.

## Author Summary

In eukaryotes, the expression of genes encoding proteins requires the action of hundreds of factors, together with the RNA polymerase II. While these factors are timely and selectively required for the expression of a given gene, little is known about their partnership upon gene expression. Our results reveal a cooperation between different types of transcription factors, namely the general transcription factor TFIIH, the cofactor PGC-1α and the deacetylase SIRT1. Such partnership is however impaired when TFIIH is mutated, as observed in Trichothiodystrophy patients that develop premature ageing. These results thus shed light on the coordinated action of factors during transcription and allow us to better understand molecular deficiencies observed in many human diseases.

## Introduction

In response to various physiological signals, selective and combined actions of a number of transcription factors modulate the expression of protein-coding genes [1]. In eukaryotes, the combinatorial use of hundreds of proteins is required for the synthesis of a single messenger RNA by the RNA Polymerase II (RNA Pol II) in association with the general transcription factors TFIIA, B, D, E, F and H [2]. Such number of protagonists requires dynamic networks to coordinate their actions during transcription. This is notably the case when a critical physiological parameter as glycaemia must be preserved within a narrow range. Indeed, to avoid the deleterious effects of hypo or hyperglycemia, the organism maintains steady circulating glucose levels, providing glucose for cells dependent on this fuel, such as neuronal and red blood cells. Apart from modulation of enzymes activity through posttranslational modifications and allosteric controls, some transcriptional regulations of rate limiting enzymes are intimately involved in maintaining blood glucose levels. Such transcriptional control is fundamental in the liver, which plays a central role in integrating signals of several cell types and multiple metabolic pathways. In particular, during starvation, in response to physiological signals like glucagon and glucocorticoids, hepatic gluconeogenic genes (such as the *phosphoenolpyruvate carboxykinase Pepck* and the *glucose-6-phosphatase G6Pase*) are regulated through the selective action of many transcription factors [3], including cAMP-responsive element binding protein (CREB) [4] in association with the CREB-regulated transcription coactivator 2 (CRTC2/TORC2) [5], CCAAT enhancer-binding protein (C/EBP) [6], Forkhead O box 1 (FOXO1) [7], [8], hepatocyte nuclear factors (especially HNF-4α) [9] and the glucocorticoid receptor (GR) [10], [11]. The transactivation mediated by these transcription factors is potentiated synergistically by different coactivators, such as the peroxisome proliferator-activated receptor-α coactivator 1 (PGC-1α). Interestingly, although its role on gluconeogenic gene remains unclear [12], [13], PGC-1α gene expression during fasting is rapidly induced in mouse liver, concomitantly with PEPCK and G6Pase up-regulation [14], [15]. Strikingly, PGC-1α activity is closely dependent to its deacetylation by the energy sensor SIRT1 (silent mating type information regulation 2 homolog 1), which is stimulated by the intracellular NAD+ and indirectly by pyruvate high concentrations related to starvation [15], [16].

Many transcription factors have been identified during the fasting-induced expression of gluconeogenic genes, but little is known about their cooperative interactions with the general transcription machinery. Among the basal transcription factors, TFIIH plays a pivotal role during transcription by interacting with different factors including nuclear receptors [17]. TFIIH, which is also intimately implicated in the nucleotide excision repair (NER) pathway, is a multienzymatic protein complex that can be resolved into two subcomplexes: the core (containing the helicase XPB, p62, p52, p44, p34 and TTDA) and the cdk-activating kinase complex (CAK, containing MAT1, CYCLIN H and the cyclin-dependent kinase CDK7). While the helicase XPD subunit, which bridges the CAK to the core of TFIIH, mainly functions in NER [18], the XPB helicase catalyzes DNA unwinding around the transcription initiation site and contributes to the promoter escape by RNA pol II [19]. In addition, TFIIH owns a kinase activity (via its CDK7 subunit) that contributes to transcription initiation by phosphorylating the C-terminal domain (CTD) of the RNA pol II largest subunit [20]. The phosphorylation by CDK7 is also required for the optimal transactivation mediated by different nuclear receptors, such as the peroxisome proliferator activated receptors PPARs [21] and the thyroid hormone receptors TR [22].

The key role played by TFIIH is illustrated by the fact that several mutations in its XPB, XPD and TTDA subunits lead to the rare autosomal recessive disorders Xeroderma Pigmentosum (XP), sometimes associated with Cockayne syndrome (XP/CS), and Trichothiodystrophy (TTD). Besides photosensitivity and skin cancers, these patients can exhibit a large spectrum of clinical abnormalities, including skeletal defects, mental retardation, immature sexual development and dwarfism. To gain more insight into these complex clinical symptoms, mouse models bearing TFIIH mutations found in patients have been generated. In particular, the TTD mouse model (having the most common XPD/R722W point mutation found in TTD patients) [23] develops a phenotype similar to what observed in patients [24] including, beside the typical dry and brittle hairs [25], progressive cachexia and hypoplasia of the adipose tissues [26]. TTD mice weight loss is not related to aberrant food uptake or intestinal malabsorption [21], and analysis of blood cell parameters of adult mice only revealed a mild normochromic anaemia and decreased levels of branched-chain amino acids that were potentially related to starvation [24]. TTD mice thus suffer from progressive failure to thrive, which is likely to be the cause of premature death, but the major cause of this failure is unknown. Although TTD has been defined at first as a DNA repair syndrome [27], transcriptional impairments may have a role in the development of TTD phenotypes [17]. In this regard, it has been shown that PPARγ dysfunction contributes to the hypoplasia of the adipose tissues [21]. However, the pleiotropic nature of the TTD phenotypes might imply combinational defects of other additional transcription factors.

To study the transcriptional defects that might occur in TFIIH related diseases, TTD mice were subjected to different fasting periods. According to the fact that the physiological adaptation to fasting requires accurate genes regulation in the liver, the hepatic response to starvation of TTD mice was particularly studied. Taken together, our results show that TTD mutation disrupts the dynamic partnership between TFIIH, SIRT1 and PGC-1α, thus impeding the PGC-1α deacetylation by SIRT1 and consequently the correct expression of gluconeogenic genes.

## Results

### Effects of food withdrawal in TTD mice

**Figure 1:**
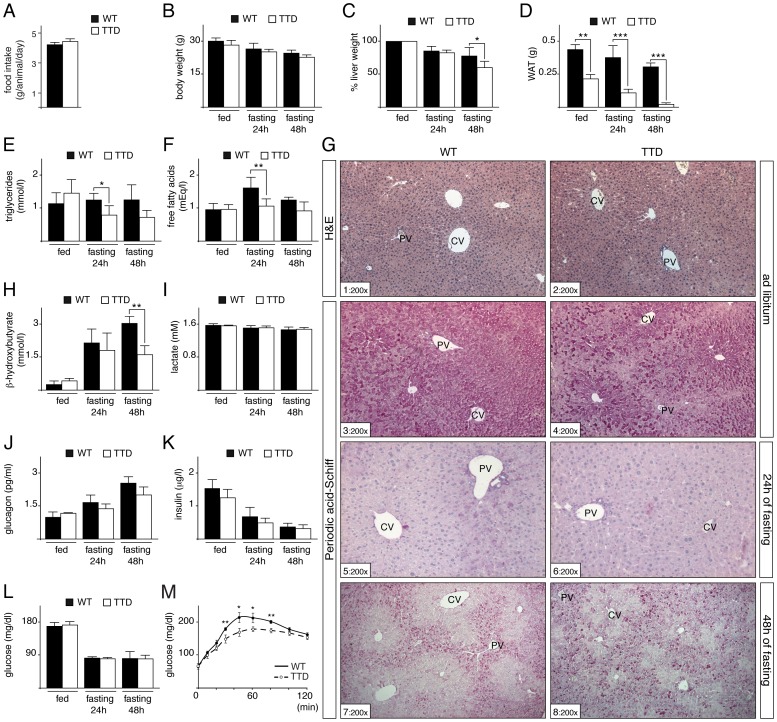
Fasting response of WT and TTD mice. (**panel A**) Daily food intake of WT (black box, n = 6) and TTD (open box, n = 6) mice during 15 days. Measurement of the body (**panel B**), liver (**panel C**) and epididymal white adipose tissue (WAT, **panel D**) weight of WT (black boxes) and TTD (open boxes) mice fed *ad libitum* or fasted for 24 h or 48 h. Values for liver weight are percentages relative to the *ad libitum* weight. Serological levels of triglycerides (**panel E**), free fatty acids (**panel F**), β-hydroxybutyrate (**panel H**), lactate (**panel I**), glucagon (**panel J**), insulin (**panel K**) and blood glucose (**panel L**) in WT (black boxes) and TTD (open boxes) fed normally or fasted for 24 h or 48 h. Error bars represent standard deviations. (**panel M**) Pyruvate tolerance tests. WT (solid curves, n = 4) and TTD (dashed curves, n = 4) mice were fasted for 16 h and injected with sodium pyruvate (2 g/Kg of body weight). The data are means ± SEM. (**panel G**) Hematoxylin & Eosin (H&E) staining of liver sections from WT and TTD mice fed normally (sections 1–2) and Periodic Acid Schiff staining of liver sections from WT and TTD mice fed normally (sections 3–4) and fasted for 24 h (sections 5–6) or 48 h (sections 7–8). PV =  Portal Vein; CV =  Central vein. Magnification is indicated at the bottom left of each section. The statistical symbols reflect significant differences between genotypes (*, p<0.05; **, p<0.01; ***, p<0.001 Student's t-test).

While TTD mice suffer from progressive failure to thrive, the young adults mice did not yet develop severe metabolic alterations when normally fed [21], [24](ref therein). Having observed that the daily food intake was identical between 3-months-old WT and TTD mice (Figure 1A), the two lines have been then subjected to different periods of fasting (24 and 48 hours). While the body weight was progressively decreased in both strains (Figure 1B), the liver weight was reduced in WT and TTD mice after 24 hours (reduction of 14.4%±6.5% in WT and 17.3%±3.6% in TTD, respectively), followed by a higher reduction in TTD mice, losing almost 40% (39.8%±9.2%) of the weight after prolonged fasting (48 hours) (Figure 1C). Different serological analyses were performed and revealed slight differences after starvation between the two strains, especially for triglycerides, free fatty acids and ketones bodies (as illustrated by β-hydroxybutyrate) (Figure 1E, F, H), which might be related to the lower fat mass already observed in TTD fed normally (Figure 1D) [21]. In parallel, fasting blood lactate levels were similar between the two lines (Figure 1I) [28], suggesting that lactate was normally utilized in TTD mice as a major substrate for glucose synthesis via gluconeogenesis. Finally, insulin and glucose, at a comparable level at fed state in WT and TTD mice, were similarly reduced during fasting (Figure 1K and 1L), while glucagon progressively increased in WT and TTD (Figure 1J). In parallel, to evaluate the gluconeogenic potential of TTD mice, pyruvate tolerance tests were undertaken (Figure 1M). After injection of the gluconeogenic substrate pyruvate, TTD mice showed significant lower plasma glucose levels at 30, 45, 60 and 80 min, after what they reached the same levels of that observed in WT mice. Taken together, these data suggest that, although able to maintain physiological glucose level, TTD mice might develop subtle gluconeogenic defects upon fasting.

Since the liver plays a central role in gluconeogenesis, we next focused our investigation on the hepatic response to fasting in TTD mice. We previously observed no significant difference in the architecture of the hepatic parenchyma (as revealed after H&E staining, Figure 1G, sections 1 and 2) between WT and TTD mice fed normally [21]. However, glycogen content, visualized through Periodic Acid-Schiff staining (sections 3–8), showed slight preferential accumulation in pericentral hepatocytes (located around pericentral veins, CV) of WT mice fed normally (section 3), while such glycogen accumulation seemed to be more pronounced in TTD livers (compare sections 3 and 4). As expected, the glycogen stores were totally depleted in WT and TTD livers after 24 h of fasting (sections 5 and 6). Afterwards, glycogen was re-accumulated in periportal hepatocytes (located around periportal veins, PV) during a prolonged fasting period (48 h, sections 7 and 8), indicating that glucose-6-phosphate was channeled towards glycogen, as previously observed in rodent livers [28], [29]. However, such *de novo* glycogen accumulation was disorganized in TTD livers, with a higher number of hepatocytes bearing strong intracellular glycogen deposits when compared to WT (compare sections 7 and 8).

### Deregulation of gluconeogenic genes in TTD liver

**Figure 2:**
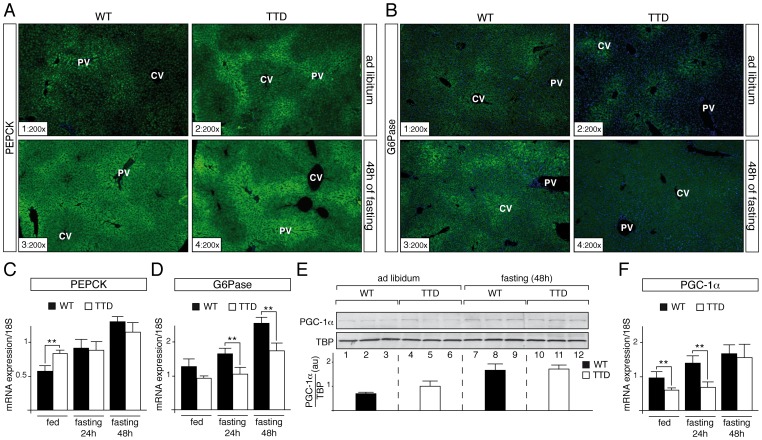
Dysregulation of gluconeogenesis-induced proteins in TTD liver. PEPCK (**panel A**) and G6Pase (**panel B**) immunostainings of liver sections from *ad libitum* (sections 1–2) and 48 h fasted (sections 3–4) WT and TTD mice. PV =  Portal Vein; CV =  Central vein. Magnification is indicated at the bottom left of each part. Expression of the hepatic fasting-induced *Pepck* (**panel C**) and *G6pase* (**panel D**) genes in WT (black boxes, n = 4) and TTD (open boxes, n = 4) fed normally or fasted for 24 h or 48 h. Results are expressed as the mean normalized to 18S RNA. (**panel E**) Western Blot analyses of PGC-1α (110 kDa) levels in the liver of three WT and three TTD fed normally (lanes 1–6) or fasted for 48 h (lanes 7–12). TBP (TATA box Binding Protein, 36 kDa) has been used as an internal control. Diagram represents the mean of the ratios between PGC-1α and TBP for each group. (**panel F**) Expression of the *Pgc-1α* gene in WT (black boxes, n = 4) and TTD (open boxes, n = 4) fed normally or fasted for 24 h or 48 h. Results are expressed as the mean normalized to 18S RNA. Error bars represent standard deviations. The statistical symbols reflect significant differences between genotypes (**, p<0.01, Student's t-test).

Knowing that PEPCK and G6Pase are two key hepatic gluconeogenic enzymes required to meet energy demands during stressful conditions like starvation [3], we analyzed their zonal distribution in TTD liver. Surprisingly, in normal feeding conditions immunohistochemical (IHC) staining of PEPCK showed a higher signal in hepatocytes located around portal vein (PV) in TTD liver (Figure 2A, sections 1–2). Prolonged fasting increased the PEPCK protein levels within the hepatic parenchyma, with a persistent higher signal in TTD when compared to WT (sections 3–4). In parallel, IHC staining of G6Pase revealed a low signal around central vein (CV) in livers of WT and TTD mice fed normally (Figure 2B, sections 1 and 2). After 48 h of fasting G6Pase protein level raised throughout the liver parenchyma of WT mice (section 3), whereas it did not increase in TTD (section 4).

Knowing that the *Pepck* and *G6pase* genes are tightly regulated at a transcriptional level [14], we analyzed the amount of their corresponding mRNA by quantitative RT-PCR. We repeatedly observed a higher amount of *Pepck* mRNA in liver of TTD mice fed *ad libitum* when compared to WT (Figure 2C), which mirrored the protein amount observed by IHC analyses (Figure 2A, sections 1–2); after fasting, the *Pepck* mRNA amount progressively increased in WT liver, whereas its accumulation occurred tardily in TTD liver, which might be related to the fact that basal levels at the feeding state were higher (Figure 2C). In parallel, the amount of the *G6pase* mRNA progressively increased in the liver of WT, while its accumulation seemed to be delayed in TTD liver (Figure 2D).

These data prompted us to study the PGC-1α coactivator, which contributes to the hepatic transcriptional activation of the *Pepck* and *G6pase* genes during starvation [14], [15]. Whereas Western Blot analyses showed an increase of the PGC-1α protein level in WT and TTD liver after 48 h of fasting (Figure 2E, compare lanes 7–9 to lanes 1–3 and lanes 10–12 to lanes 4–6, respectively), quantitative RT-PCR analyses revealed that the mRNA amount of the fasting-induced *Pgc-1α* gene was delayed in TTD liver when compared of that observed in WT (Figure 2F). Taken together, these data suggest that defective expression of gluconeogenic genes occurs during fasting in TTD liver.

### Defective recruitment of PGC1-α in TTD hepatocytes

**Figure 3:**
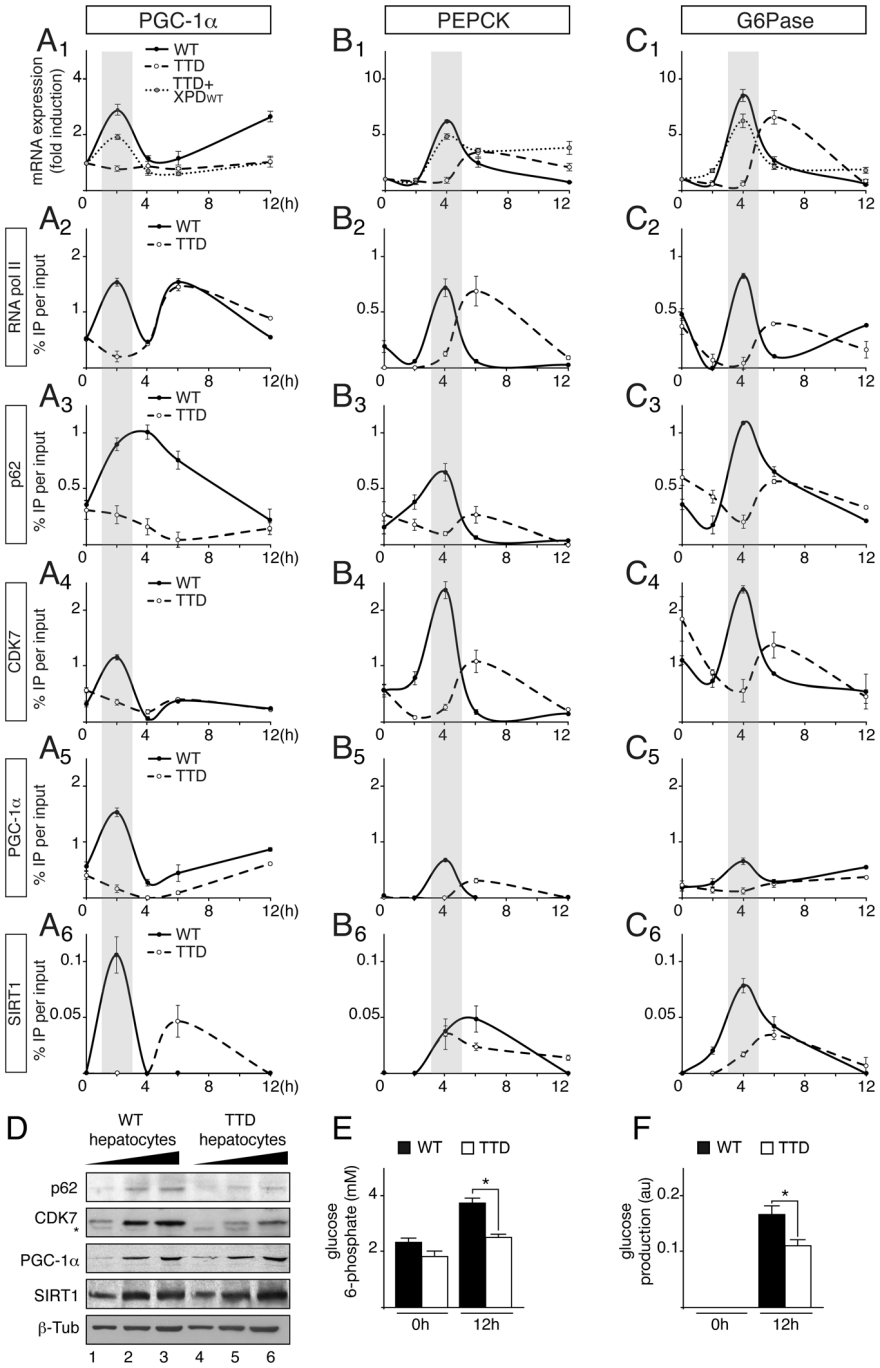
Defective recruitments of transcription factors on the promoter of gluconeogenic genes in TTD hepatocytes. Expression of *Pgc-1α* (**panel A1**) *Pepck* (**panel B1**) and *G6Pase* (**panel C1**) genes in WT (solid curves), TTD (dashed curves) and TTD overexpressing XPDwt (dotted curves) hepatocytes after pyruvate treatment. The results are presented as n-fold induction relative to non-treated cells. Recruitment of RNA pol II, p62, CDK7, PGC-1α and SIRT1 on the proximal promoter of PGC-1α (**panels A2 to A6**), PEPCK (**panels B2 to B6**) and G6Pase (**panels C2 to C6**) in WT (dotted curves) and TTD (dashed curves) hepatocytes. The results of three independent experiments are presented as percentage of DNA immunoprecipitated relative to the input. The shaded areas underline the concomitant recruitments of the transcription factors with the expression profile of the target genes in WT hepatocytes. (**panel D**) Western blot analyses of TFIIH, illustrated by its p62 (62 kDa) and CDK7 (39 kDa) subunits, PGC-1α (110 kDa) and SIRT1 (110 kDa) with increasing amounts of whole cell extracts isolated from WT (lanes 1–3) and TTD (lanes 4–6) hepatocytes. β-tubulin (β-Tub, 50 kDa) has been used as an internal control. * indicates unspecific band. Measurement of intracellular glucose 6-phosphate (**panel E**) and glucose output (**panel F**) levels from WT (black boxes) and TTD (open boxes) hepatocytes after 0 and 12 hours of pyruvate treatment. Values represent the means ± SEM. The statistical symbols reflect significant differences between genotypes (*, p<0.05, Student's t-test).

To accurately dissect the transcriptional response occurring in TTD liver during fasting, hepatocytes were immortalized from WT and TTD mouse embryonic livers and then treated with medium devoid of glucose and supplemented with pyruvate, forskolin and glucagon, a treatment (referred for convenience pyruvate treatment) known to stimulate the gluconeogenic pathway [15]. Quantitative RT-PCR showed that the *Pgc-1α* gene was weakly induced 2 hours post-treatment in TTD hepatocytes when compared of that observed in WT hepatocytes (Figure 3A1). The expression of the PGC-1α-dependent *Pepck* and *G6pase* genes was lower and delayed in TTD cells when compared to their induction in WT hepatocytes (Figure 3B1 and C1, respectively). Remarkably, transfection of XPDwt in TTD hepatocytes restored the expression profiles of the *Pgc-1α*, *Pepck* and *G6Pase* genes similarly to that observed in normal cells (Figure 3A1, B1 and C1), suggesting that the mutation in the *xpd* gene promoted the defective expression of gluconeogenic genes observed in TTD hepatocytes. It is worthwhile to notice that the expression of PGC1-α target genes involved in fatty acid oxidation, such as *Cpt1a* (Carnitine palmitoyltransferase Ia) and *Mcad* (Medium-chain acyl-CoA dehydrogenase) was also disrupted in TTD hepatocytes (Figure S1A).

Chromatin Immunoprecipitation (ChIP) assays then showed that RNA pol II and TFIIH (visualized by the presence of its p62 and CDK7 subunits) were recruited 2 hours post-treatment at the PGC-1α promoter (Figure 3, panels A2 to A4) and 4 h post-treatment at the PEPCK and G6Pase promoters (panels B2–B4 and C2–C4, respectively) in WT hepatocytes, matching the expression profile of the corresponding genes. On the contrary, RNA pol II and TFIIH recruitments were altered and delayed in TTD hepatocytes. Interestingly, we also noticed that the recruitment of PGC-1α was disrupted on its own promoter as well as on that of PEPCK and G6Pase (panels A5, B5 and C5). As a consequence, the recruitment profiles of transcription factors that are activated by PGC-1α, such as HNF-4α [9], FOXO1 [30] and CREB [31], were altered in TTD hepatocytes (Figure S1B). It is worthwhile to notice that the protein amounts of HNF-4α, FOXO1 and CREB were similar in WT and TTD hepatocytes (Figure S1C) and the nuclear localization as well as the acetylation status of FOXO1 was not disrupted in TTD hepatocytes (Figure S1D). Finally, the recruitment of the deacetylase SIRT1, which activates PGC-1α [15], was also disrupted in TTD hepatocytes (Figure 3, panels A6, B6 and C6). Strikingly, Western Blots analyses revealed that the PGC-1α and SIRT1 protein amounts were similar in WT and TTD hepatocytes (Figure 3D), suggesting that their defective recruitments on the promoter of target genes in TTD were not due to lower protein amounts. Nonetheless, we also noticed that the amount of TFIIH subunits (such as p62 and CDK7) was reduced in TTD cells (Figure 3D) [32].

Taken together, these data show that the expression of PGC-1α is altered in TTD hepatocytes, resulting in a defective regulation of PGC-1α-targeted gluconeogenic genes. As a consequence, intracellular glucose 6-phosphate (Figure 3E) and glucose output (Figure 3F) were reduced in TTD hepatocytes after 12 h of pyruvate treatment.

### Partnerships between TFIIH, PGC-1α and SIRT1

**Figure 4:**
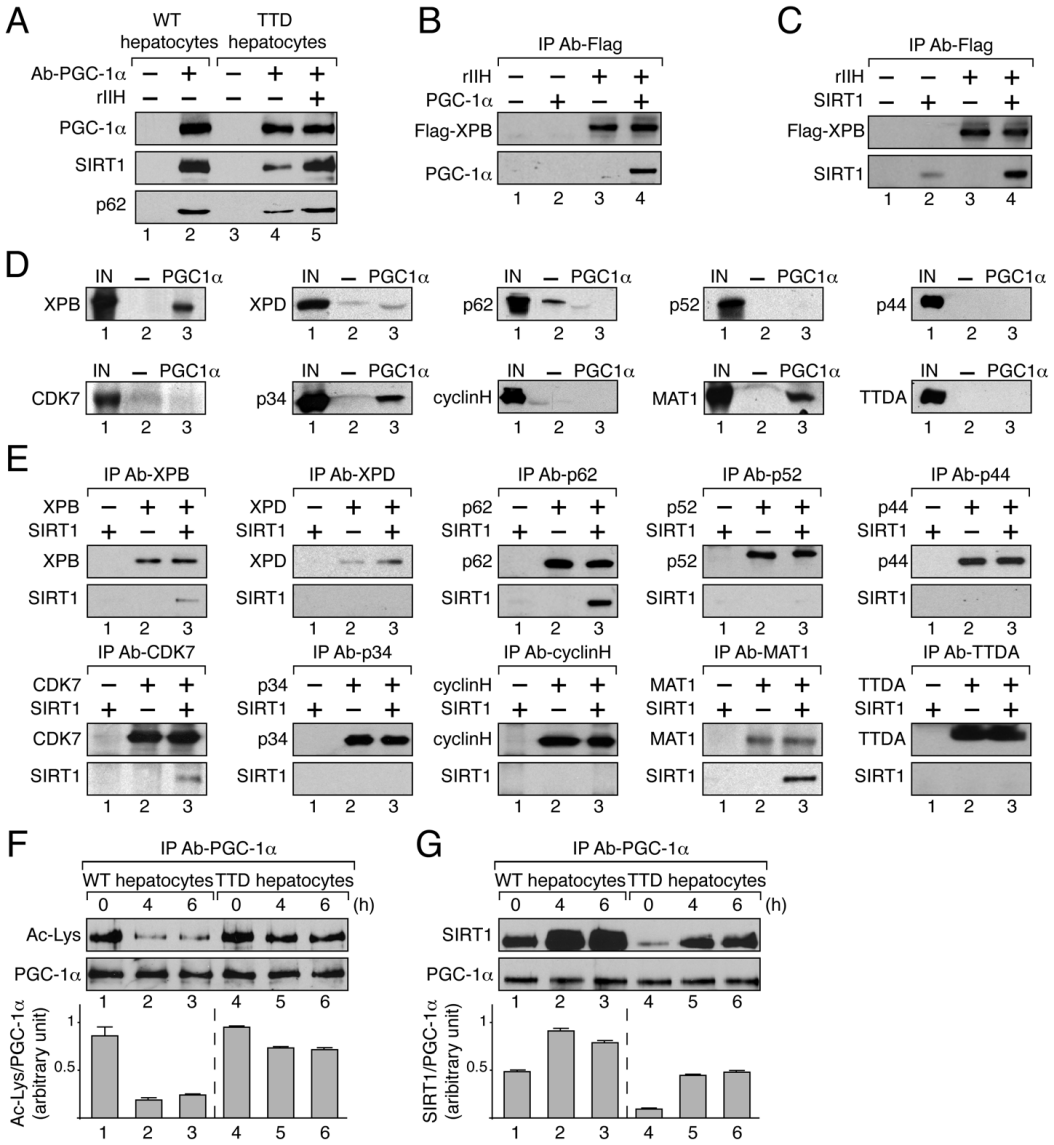
TFIIH influences PGC-1α deacetylation by SIRT1 by interacting with both. (**panel A**) After immunoprecipitation of PGC-1α from nuclear extracts of WT (lanes 1–2) and TTD (lanes 3–5) hepatocytes, co-immunoprecipitated proteins were visualized by western blots with antibodies raised against PGC-1α (110 kDa), SIRT1 (110 kDa) and the p62 subunit (62 kDa). TTD nuclear extract was supplemented with recombinant TFIIH (rIIH, lane 5). (**panel B**) When indicated (+), GST-PGC-1α purified from bacteria (130 kDa) was incubated with lysate of Sf9 cells overexpressing TFIIH (rIIH). After immunoprecipitation with an anti Flag-Tag antibody (that recognized the flagged XPB subunit, 89 kDa), the bound proteins were visualized by western blots using antibodies raised against PGC-1α and XPB. (**panel C**) Purified SIRT1 (110 kDa) was incubated with lysate of Sf9 cells overexpressing TFIIH (rIIH). Immunoprecipitations were performed as described panel B. The bound proteins were visualized by western blots using antibodies raised against SIRT1 and XPB. (**panel D**) In vitro pull-down assays were performed with GST alone (-, 26 kDa, lanes 2) or GST-PGC-1α (PGC-1α, 130 kDa, lanes 3) incubated with Sf9 cell extracts overexpressing separately each subunit of TFIIH. The bound proteins were visualized by western blots using antibodies directed against each TFIIH subunit. As a reference, the input lanes (IN, lanes 1) represent 10% of the total volume of extract used for each incubation. (**panel E**) Purified SIRT1 was incubated with Sf9 cell extracts overexpressing separately each TFIIH subunit. Immunoprecipitations (IP) were done using antibodies directed against the TFIIH subunits. The bound proteins were revealed by western blots. (**panel F**) Deacetylation profile of PGC-1α in WT (lanes 1–3) and TTD (lanes 4–6) hepatocytes after different times of pyruvate treatment (0, 4 and 6 hours). After immunoprecipitation with specific antibodies (IP Ab-PGC-1α), PGC-1α acetylation has been visualized by western blots with anti-acetyl lysine antibodies. Graph depicts the ratio of acetyl-Lysine (Ac-Lys)/PGC-1α western blots signals. (**panel G**) PGC-1α was immunoprecipitated with specific antibodies (IP Ab-PGC-1α) from nuclear extracts of WT (lanes 1–3) and TTD (lanes 4–6) hepatocytes after different times of pyruvate treatment (0, 4 and 6 hours). Co-immunoprecipitated proteins were visualized by western blots with anti-PGC-1α and -SIRT1 antibodies. Graph depicts the binding ratio between SIRT1 and PGC-1α.

Since TFIIH and PGC-1α have in common the ability to coactivate some nuclear receptors [22], [33], [34], and the activation by the latter requires its deacetylation by SIRT1 [15], [16], we investigated the partnerships that might exist between TFIIH, SIRT1 and PGC-1α. Having observed that TFIIH mutation disrupted PGC-1α and SIRT1 recruitment at the promoter of target genes (Figure 3A5–6, B5–6 and C5–6), we investigated whether these factors interacted with each other. Antibodies directed towards PGC-1α co-immunoprecipitated SIRT1 as well as TFIIH (illustrated by the presence of its p62 subunit) from WT hepatocytes nuclear extracts (Figure 4A, lane 2). Strikingly, these co-immunoprecipitations were reduced in TTD hepatocytes extracts (compare lanes 2 and 4 in Figure 4A). However, supplementation of TTD nuclear extracts with highly purified recombinant TFIIH (rIIH) potentiated the binding of SIRT1 to PGC-1α to a level similar to that observed in WT (Figure 4A, lanes 4 and 5). In a second set of assays, we observed that rIIH co-immunoprecipitated with purified PGC-1α (Figure 4B) [35] and SIRT1 (Figure 4C). PGC-1α specifically interacted with recombinant XPB, p34 and MAT1 subunits of TFIIH (Figure 4D), while SIRT1 co-immunoprecipitated with XPB, p62, cdk7 and MAT1 (Figure 4E).

PGC-1α acetylation profile was then analyzed in WT and TTD hepatocytes after pyruvate treatment. Western Blots revealed that the immunoprecipitated PGC-1α was deacetylated until 6 h post-treatment in WT hepatocytes (Figure 4F, lanes 1–3) concomitantly to a higher co-immunoprecipitation of SIRT1 (Figure 4G, lanes 1–3), thereby allowing the recruitment of PGC-1α on targeted promoters (Figure 3A5, B5 and C5). On the contrary, in TTD hepatocytes PGC-1α acetylation remained relatively high (Figure 4F, lanes 5–6), which was in accordance with the lower co-immunoprecipitation of SIRT1 (Figure 4G, lanes 5–6) and with the weaker recruitment of PGC-1α on targeted promoters (Figure 3A5, B5 and C5). Taken together these results show that PGC-1α and SIRT1 both interact with TFIIH, and suggest that the reduced amount of TFIIH in TTD hepatocytes affects the partnership between SIRT1 and PGC-1α and consequently PGC-1α deacetylation.

### Dynamic connection between TFIIH, PGC-1α and SIRT1

**Figure 5:**
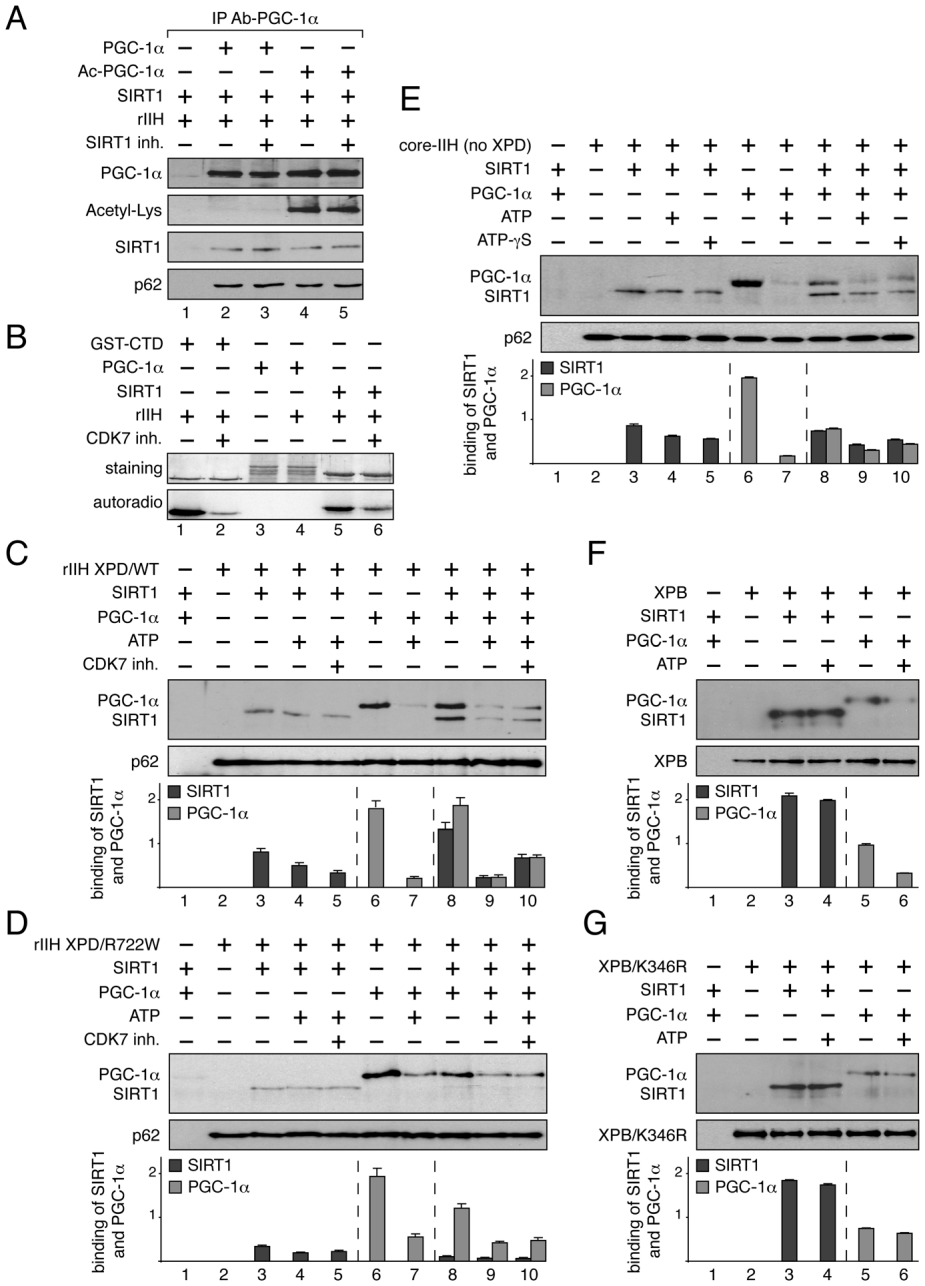
Dynamic partnership between TFIIH, PGC-1α and SIRT1. (**panel A**) SIRT1 and TFIIH bind to PGC-1α independently to its acetylation status. After immunoprecipitation with specific antibodies (IP Ab-PGC-1α), non acetylated (PGC-1α, lanes 2–3) and acetylated PGC-1α (Ac-PGC-1α, lanes 4–5) were incubated with purified SIRT1 and recombinant TFIIH (rIIH). When indicated (+), specific SIRT1 inhibitor (SIRT1 inh., 10 µM) was also added. Co-immunoprecipitated proteins were visualized by western blots with anti-PGC-1α, -acetyl-Lysine, -p62 and -SIRT1 antibodies. **(panel B)** *In vitro* phosphorylation of SIRT1. When indicated (+), GST-C-terminal domain of the largest subunit of the RNA pol II (GST-CTD, 90 kDa) (used as positive control, lanes 1–2) [53], PGC-1α (130 kDa, lanes 3–4) and SIRT1 (110 kDa, lanes 5–6) were incubated with recombinant TFIIH (rIIH) in the presence of [γ-^32^P] ATP and CDK7 inhibitor (CDK7 inh.). Coomassie blue staining gel (top panels) and autoradiography (bottom panels) of the incubated fractions are shown. **(panels C–G)** When indicated (+), SIRT1 (110 kDa), PGC-1α(130 kDa, ATP (100 nM), CDK7 inhibitor (CDK7 inh.) and non-hydrolyzable ATP analog (ATP-γS, 100 nM) were incubated with either immunoprecipitated recombinant TFIIH with WT XPD subunit (rIIH XPD/WT, **panel C**), recombinant TFIIH with mutated XPD (rIIH XPD/R722W, **panel D**), core-TFIIH without XPD (**panel E**), XPB subunit (**panel F**) or mutated XPB bearing the point mutation K346R (XPB/K346R, **panel G**). Co-immunoprecipitated proteins were visualized by western blots using anti-SIRT1, -PGC-1α, -p62 and -XPB antibodies. Graphs depict the binding of PGC-1α and SIRT1.

The connection between TFIIH, PGC-1α and SIRT1 was further investigated. We first evaluated whether the acetylation status of PGC-1α influenced its interaction with TFIIH and SIRT1 (Figure 5A). Non acetylated and acetylated PGC-1α were immunoprecipitated from WT hepatocytes and were incubated with SIRT1 and recombinant TFIIH (rIIH). Our results revealed that SIRT1 as well as TFIIH (illustrated by the presence of its p62 subunit) were able to co-immunoprecipitate with PGC-1α regardless of its acetylation status (lanes 2 and 4). Furthermore, addition of a specific SIRT1 inhibitor (EX-527, 10 µM) [36], [37] did not affect the TFIIH/PGC-1α/SIRT1 complex, whichever the PGC-1α acetylation status (lanes 3 and 5).

Having observed that TFIIH (via its CDK7 kinase subunit) phosphorylated in vitro SIRT1 and not PGC-1α (Figure 5B), recombinant TFIIH containing wild-type XPD subunit (rIIH-XPD/WT) was immunoprecipitated (with antibodies directed against the XPB subunit) and incubated with SIRT1 and/or PGC-1α in the presence or absence of 100 nM ATP (Figure 5C). We firstly noted that the interaction between SIRT1 and rIIH XPD/WT was not modified upon addition of either ATP (lane 4) or a CDK7 kinase inhibitor (CDK7 inh., lane 5), suggesting that the CDK7-mediated phosphorylation of SIRT1 did not influence its binding to TFIIH. On the contrary, addition of ATP disrupted the interaction of PGC-1α to rIIH XPD/WT (lanes 6–7). While the presence of PGC-1α potentiated the binding of SIRT1 to the immunoprecipitated rIIH XPD/WT (lane 8), we observed that addition of ATP promoted the release of PGC-1α and therefore of SIRT1 (lane 9). Such effect was not related to the CDK7 kinase activity, since the addition of CDK7 inhibitor did not affect the release of SIRT1 as well as of PGC-1α (lane 10).

Knowing that the TTD mutated form XPD/R722W affects the integrity of TFIIH by weakening the binding of the CAK subcomplex to the core of TFIIH [18], [38], we next evaluated the consequences of such mutation on the TFIIH/SIRT1/PGC-1α complex formation (Figure 5D). Whereas the binding of SIRT1 alone to the immunoprecipitated rIIH XPD/R722W was not modified even in presence of ATP (lane 3–5), we observed that ATP modulated the binding of PGC-1α (lanes 7 and 9). Furthermore, contrary to that observed with rIIH XPD/WT, the binding of SIRT1 to rIIH XPD/R722W was not enhanced by the presence of PGC-1α (lane 8), suggesting that the integrity of TFIIH is crucial for the optimal binding of SIRT1.

The core of TFIIH without XPD and the CAK subcomplex (core-IIH no XPD) was then immunoprecipitated using antibodies directed against XPB, and incubated with SIRT1 and/or PGC-1α (Figure 5E). Although SIRT1 and PGC-1α bound the core-IIH (lanes 3 and 6, respectively), the binding of PGC-1α in the presence of SIRT1 was reduced when compared to that observed with rIIH XPD/WT (Figure 5C, lane 8), suggesting that XPD and the CAK subcomplex are required for accurate binding of PGC-1α. In parallel, the binding of SIRT1 to the core-IIH was not strongly potentiated by the presence of PGC-1α (lane 8). Interestingly, while ATP promoted the release of PGC-1α from the immunoprecipitated complex (lane 7), it did not modify the binding of SIRT1 (lane 4), suggesting that the ATP-dependent release of PGC-1α did not involve the CAK subcomplex. Furthermore, non-hydrolysable ATP analog (ATP-γS, 100 nM) promoted the release of PGC-1α (and therefore of SIRT1) from the core-TFIIH (lane 10).

Having noticed that both PGC-1α and SIRT1 interacted with the XPB ATP-binding subunit of TFIIH (Figure 4D and 4E, respectively), we incubated these three proteins in the presence of ATP (Figure 5F). After immunoprecipitation of XPB, we observed that ATP promoted the release of PGC-1α (lanes 6) without affecting the binding of SIRT1 (lane 4). Our data prompted us to immunoprecipitate a mutated form of XPB that does not bind ATP (XPB/K346R, Figure 5G) [39], [40], [41]. While SIRT1 and PGC-1α interacted with this mutated form of XPB (lanes 3 and 5, respectively), no release of PGC-1α was observed in the presence of ATP (lane 6). Taken together, these results suggest that the binding of ATP to XPB influences the PGC-1α release without affecting the interaction with SIRT1.

## Discussion

The production of glucose during fasting requires an extensive use of metabolites, including lactate, amino acids, triglycerides and ketone bodies. Although serological analyses from fasted TTD mice revealed slight reductions when compared to WT for triglycerides, free fatty acids and ketones bodies (Figure 1E, F, H) [21], TTD mice seemed to be able to cope with prolonged fasting periods by maintaining glucose at a level similar to that observed in normal mice (Figure 1L). However, such maintenance in front of a stressful condition resulting from food deprivation was accompanied by failures in the liver. In particular, disruption was quickly observed during pyruvate tolerance tests (Figure 1M), which implies defects in the liver and certainly in other peripheral tissues, such as the adipose tissues. Defect in different metabolic processes might be implicated, such as lipid uptake, mitochondrial function and gluconeogenic pathway. Furthermore, the *de novo* hepatic accumulation of glycogen occurring after longer fasting period was also disrupted in TTD (Figure 1G). Although the gluconeogenic enzymes defects observed in TTD livers (Figure 2) might contribute to the glycogen accumulation abnormalities, further investigations should be performed in order to claim such assertion.

To maintain blood glucose levels in conditions of food deprivation, a precise regulation of the hepatic genes that control glucose production is required. However, the accurate and timely expression of the gluconeogenic genes *Pepck* and *G6pase* was clearly disrupted in TTD liver (Figure 2C, D), which can therefore explain the abnormal amount of PEPCK and G6Pase observed after fasting (Figure 2A, B). Although the phenotypes observed in TTD might result from pleiotropic effects due to the dysfunctions of various transcription factors, the deregulation of the gluconeogenic genes seems at least in part to concern PGC-1α, a coactivator whose induction is also delayed in fasted TTD (Figure 2F), and this raises the question on the role of the mutated TFIIH in such defect. By weakening the overall structure of TFIIH and its cellular concentration (Figure 3D) [18], [38], it seems that the XPD/R722W mutation prevents the capacity of TFIIH to correctly interact with PGC-1α and SIRT1 (Figure 4A and 5C) and to promote the PGC-1α deacetylation by SIRT1 (Figure 4F). Thus, PGC-1α recruitment is impaired on the promoter of PEPCK and G6Pase (Figure 3 B5 and C5, respectively), having as a consequence the alteration of the concomitant recruitment of transcription factors such as HNF-4α, FOXO1 and CREB (Figure S1B). Besides the fact that the integrity of TFIIH is essential for the PGC-1α coactivation function, the partnership of TFIIH with SIRT1 and PGC-1α also requires an ATP-dependent dynamic process via the XPB subunit (Figure 5F). Interestingly, the helicase XPB subunit is known to harbor conformational change in the presence of DNA, which is facilitated by ATP hydrolysis, leading to a closed and stable XPB/DNA complex [41], [42]. It seems increasingly clear that conformational changes in XPB resulting from the binding and/or the hydrolysis of ATP would allow new connections with DNA and proteins.

**Figure 6:**
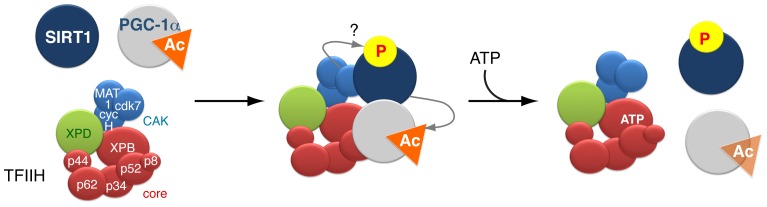
Model of the dynamic partnership between TFIIH, PGC-1α and SIRT1. SIRT1 and PGC-1α physically interact with various subunits of the TFIIH complex: SIRT1 interacts with XPB, p62, cdk7 and MAT1, while PGC-1α interacts with XPB, p34 and MAT1. SIRT1 binds to TFIIH alone, but its interaction is reinforced by the presence of PGC-1α. The simultaneous interaction between TFIIH, PGC-1α and SIRT1 suggests that TFIIH might contribute to the PGC-1α deacetylation by SIRT1. Such assumption is supported by the fact that i) the integrity of TFIIH is crucial for the optimal binding of PGC-1α and SIRT1 and ii) the PGC-1α deacetylation is disrupted by XPD mutation (such as XPD/R722W) that affects the integrity of TFIIH. In parallel, the CDK7 kinase of TFIIH targets SIRT1, but the function of such phosphorylation(s) remains elusive. Finally, the binding of ATP to the XPB subunit of TFIIH influences the release of PGC-1α, which in turn affects the binding of SIRT1.

Taken together, our observations highlight the fine and dynamic relationships that exist between the basal transcription machinery, the transcription factors and their cofactors, which allow the expression of protein coding genes at the right time and in the right amount. Previous studies showed that TFIIH participates to the transactivation mediated by different nuclear receptors by phosphorylating them [21], [22], [43]. Interestingly, the CDK7 kinase subunit of TFIIH also phosphorylates SIRT1 but not PGC-1α, at least in vitro (Figure 4A). Whereas the analysis of truncated forms of SIRT1 suggested that the catalytic and the C-terminal part of SIRT1 might be phosphorylated by the CDK7 kinase, we failed to identify the targeted residues. Interestingly, different phosphorylated sites have been already found in SIRT1 [44], some of them promoting the deacetylation activity of SIRT1 [45]. Although the role of the SIRT1 phosphorylation by TFIIH is far from being established (Figure 6), the finding of a dynamic connection between TFIIH and SIRT1 remains particularly relevant, since SIRT1 plays a critical role in health maintenance and metabolic stress responses [46], [47]. In particular, it has been shown that, while the accumulation of DNA damage caused by oxidative stress contributes to human tissue ageing, strong evidence supports a role for SIRT1 in oxidative stress response by deacetylating transcription factors that regulate the expression of stress response genes [48], [49]. Furthermore, recent data suggest that the transcriptional arrest following UV irradiation in cells bearing XPD mutations associated to the combined XP/CS syndrome results from an active and persistent heterochromatinization process mediated by SIRT1 [50]. Therefore, in addition to further understand the synergistic action of factors during gene expression, our results allow us to better apprehend the etiology of human diseases during which transcriptional mechanisms may be impaired.

## Materials and Methods

### Ethic statement

This study was performed with the agreement of the French Ministry of Higher Education and Research (permit number n°6754). Furthermore, our study has been followed by the Ethical committee of the Institute, which is registered by the French National Committee for Ethics in Animal Experimentation n°17. This study has been realized in the accredited animal house of the Institute, in compliance with European guidelines. Euthanasia using carbon dioxide has been realized on adult mice. Every effort was made to minimize suffering.

### Animal studies

The generation of the TTD mouse line XPD/R722W has been previously described [24]. Three-month-old WT and TTD males used in our experiments share identical genetic background (100% C57BL/6) and are littermates. Mice were either fed a standard chow with 5% (w/w) fat content (R03 breeding diet, UAR, Villemoisson, France) or fasted for the indicated periods. For histological analyses, liver fragments were fixed in 4% formaldehyde for 24 h prior to paraffin embedding. Liver sections were 5 µm thick, and were stained according to the European Mouse Phenotyping Resource of Standardised Screens (http://www.eumorphia.org). Liver fragments were also frozen for RNA and proteins extraction. Blood glucose levels were measured with One Touch Ultra Glucose meter (LifeScan Inc, Milpitas, California). Triglycerides, free fatty acids and β-hydroxybutyrate measurement from serum were performed with an OLYMPUS AU-400 automated laboratory workstation. Insulin and glucagon were measured using Millipore Milliplex Kit and Glucagon quantikine Elisa kit (R&D Systems Europ), respectively. Lactate and Pyruvate levels were measured using Abcam kits ab65331 and ab65342, respectively. For the Pyruvate Tolerance Tests, overnight fasted (16 h) mice were injected IP with pyruvate (2 g/kg), and the glucose level was measured before and after injection at the different time points.

### Immunohistochemistry

Immunostainings on liver sections (5 µm) were performed using polyclonal anti-G6Pase (ab83690, Abcam) and anti-PEPCK (sc-74823, Santa Cruz) antibodies. As secondary antibody, Alexa Fluor Goat Anti-Rabbit IgG (Invitrogen) diluted 1∶200 was used. Counterstaining was performed with DAPI.

### Cell culture and reagents

Hepatocytes were isolated from liver tissues derived from E14.5-murine WT and TTD embryos as previously described [51], [52]. After careful selection, WT and TTD hepatocytes were grown in Dulbecco's modified Eagle medium (DMEM) containing 10% of fetal calf serum (FCS), 40 µg/ml gentamicin, 1x nonessential amino acid solution (Fischer Scientific), 2 mM L-glutamine (Invitrogen), 1x Insulin-Transferin-Selenium solution (ITS, Invitrogen), 0,1 µg/ml dexamethasone (Sigma Aldrich) and antibiotic/antimycotic solution (Invitrogen). Before stimulation of the gluconeogenesis pathway, cells were preincubated with 1 g/l glucose and red phenol-free medium containing 2,5% charcoal treated FCS and ITS for 16 hr. Hepatocytes were then treated in absence of glucose with 1 mM pyruvate, 10 µM Forskolin (Sigma Aldrich) and 150 nM glucagon (Sigma Aldrich). 1×10^6^ harvested cells were used to measure intracellular Glucose 6-Phosphate and NAD and NADH levels (using Abcam assay kits ab83426 and ab65348, respectively). Glucose output in the medium was measured with glucose assay kit (Abcam ab65333). Hepatocytes were transiently transfected with 1 µg of PGC-1α expression vector using the transfection reagent JetPEI (Polyplus Transfection); the cells were harvested at 72 h posttransfection. To potentiate the PGC-1α acetylation, WT hepatocytes were cotransfected with p300 and treated with nicotinamide (10 mM) for 16 h before harvesting [16].

### Retrotranscription and real-time quantitative PCR

Total RNAs (2 µg) were reverse-transcribed with Moloney murine leukemia virus RT (Invitrogen) using random hexanucleotides. Real-time quantitative PCR was done using the “FastStart DNA Master SYBR Green” kit and the Lightcycler apparatus (Roche Diagnostic). The mRNA levels of interest were normalized to the 18S RNA amount, which was not affected by the different fasting periods (in the liver) and the pyruvate treatment (in the hepatocytes).

### Chromatin Immunoprecipitation (ChIP)

ChIP experiments were performed as previously described [21]. Primers were designed to amplify the proximal promoter region of PEPCK (−300 to +13), G6Pase (−122 to +54) and PGC1α (−134 to +19).

### Antibodies

Monoclonal antibodies against the TFIIH subunits, RNA pol II, GST-Tag and Flag-Tag were produced at the IGBMC. Antibodies against PEPCK (sc-74823, Santa Cruz SC), G6Pase (Ab83690, AB Cam LTD), PGC-1α (4C1.3, Calbiochem; AB3242, Millipore), SIRT1 (2028, Cell Signaling Technology CST and 3H10.2 Millipore, for murine and human SIRT1, respectively), HNF-4α (C-19, SC), FOXO1 (L27, CST), acetylated FOXO1 (D-19, SC), CREB (48H2, CST), β-tubulin (KMX-1, Millipore) and acetylated lysine (9441, SCT) were purchased.

### Plasmids and construction of PGC-1α and SIRT1

Full-length human SIRT1 and mouse PGC-1α were obtained from Addgene (plasmid #13735 and #1026, respectively). Flag-PGC-1α was overexpressed and immunoprecipitated from cells. Truncated PGC-1α (36-797 amino acids, for solubility reasons) [33], was cloned into the bacterial expression vector pGEX-4T3 and purified via the GST-Tag to perform in vitro analyses.

### *In vitro* kinase assays

His-SIRT1, GST-PGC-1α and GST-CTD proteins were produced in E. Coli strain BL21 and purified on either NI-NTA agarose (Qiagen) or glutathione column (Thermo Scientific). Equal amounts (1 µg) of recombinant proteins were incubated with highly purified TFIIH (rIIH) in the presence of [γ-^32^P] ATP (0.14 µM).

### Co-immunoprecipitation assays

Sf9 cells were infected with baculoviruses encoding either each Flag-subunit of TFIIH separately or all subunits together to produce an entire TFIIH. Whole extracts from infected cells were incubated with antibody against the Flag-Tag to immunoprecipitate either the entire TFIIH (containing only the Flag-tagged XPB subunit) or each Flag-tagged subunit individually. After incubation with His-SIRT1 and/or GST-PGC-1α during 2 h at 4°C and extensive washings (400 mM KCl), bound protein were resolved by SDS-PAGE and revealed by immunoblotting.

## Supporting Information

Figure S1 (**panel A**) Expression of *Cpt1a* (Carnitine palmitoyltransferase Ia) and *Mcad* (Medium-chain acyl-CoA dehydrogenase) genes in WT (solid curves) and TTD (dashed curves) hepatocytes after pyruvate treatment. The results are presented as n-fold induction relative to non-treated cells. (**panel B**) Recruitment of HNF-4α, FOXO1 and CREB on the proximal promoter of PGC-1α (left column), PEPCK (middle column) and G6Pase (right column) in WT (dotted curves) and TTD (dashed curves) hepatocytes. The results are presented as percentage of DNA immunoprecipitated relative to the input. (**panel C**) Western blot analyses of HNF-4α, FOXO1 and CREB with increasing amounts of whole cell extracts isolated from WT (lanes 1–3) and TTD (lanes 4–6) hepatocytes. β-tubulin (β-Tub, 50 kDa) has been used as an internal control. (**panel D**) Whole cell (WCE) and nuclear (NE) extracts isolated from WT (lanes 1–3) and TTD (lanes 4–6) hepatocytes were used to analyse by western blots the acetylated form of FOXO1 and its nuclear translocation after 0, 6 and 12 h of pyruvate treatment. TBP (36 kDa) has been used as an internal control.
(TIF)
