# Supplementary figures and images for: Dynamic Partnership between TFIIH, PGC-1α and SIRT1 Is Impaired in Trichothiodystrophy
Source: PLoS Genet. 2014 Oct 23;10(10):e1004732. doi: 10.1371/journal.pgen.1004732 (PMC4207666; doi:10.1371/journal.pgen.1004732)

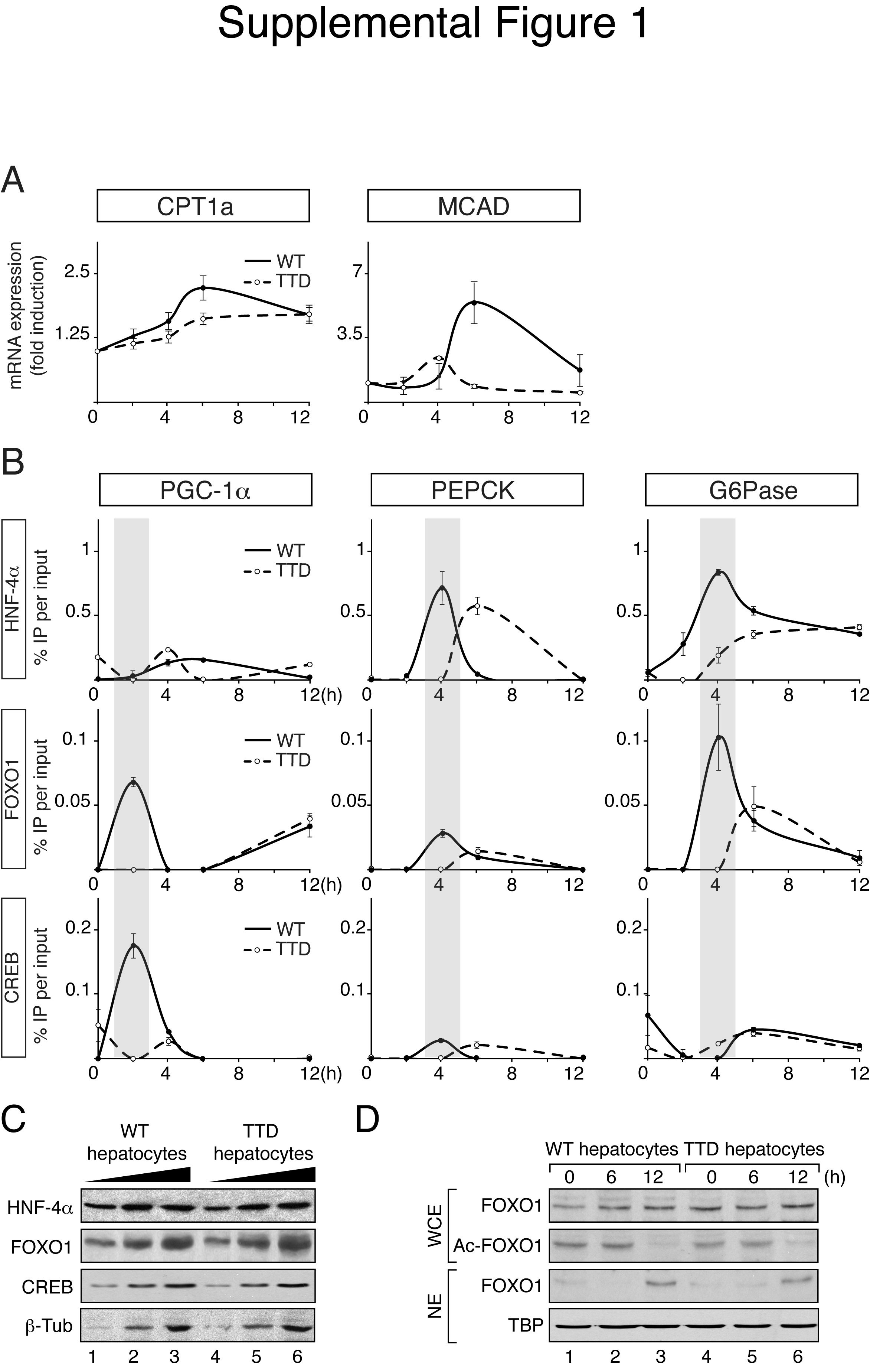

Supplement: Figure S1 — (panel A) Expression of Cpt1a (Carnitine palmitoyltransferase Ia) and Mcad (Medium-chain acyl-CoA dehydrogenase) genes in WT (solid curves) and TTD (dashed curves) hepatocytes after pyruvate treatment. The results are presented as n-fold induction relative to non-treated cells. (panel B) Recruitment of HNF-4α, FOXO1 and CREB on the proximal promoter of PGC-1α (left column), PEPCK (middle column) and G6Pase (right column) in WT (dotted curves) and TTD (dashed curves) hepatocytes. The results are presented as percentage of DNA immunoprecipitated relative to the input. (panel C) Western blot analyses of HNF-4α, FOXO1 and CREB with increasing amounts of whole cell extracts isolated from WT (lanes 1–3) and TTD (lanes 4–6) hepatocytes. β-tubulin (β-Tub, 50 kDa) has been used as an internal control. (panel D) Whole cell (WCE) and nuclear (NE) extracts isolated from WT (lanes 1–3) and TTD (lanes 4–6) hepatocytes were used to analyse by western blots the acetylated form of FOXO1 and its nuclear translocation after 0, 6 and 12 h of pyruvate treatment. TBP (36 kDa) has been used as an internal control. (TIF) [file pgen.1004732.s001.tif]
